# Seasonal flooding decreases fruit‐feeding butterfly species dominance and increases spatial turnover in floodplain forests of central Amazonia

**DOI:** 10.1002/ece3.9718

**Published:** 2023-01-06

**Authors:** Isabela Freitas Oliveira, Fabricio Beggiato Baccaro, Fernanda P. Werneck, Torbjørn Haugaasen

**Affiliations:** ^1^ Programa de Pós‐Graduação em Ecologia Instituto Nacional de Pesquisas da Amazônia – INPA Manaus Brazil; ^2^ Ecosystem Modeling, Center for Computational and Theoretical Biology (CCTB) University of Würzburg Würzburg Germany; ^3^ Faculty of Environmental Sciences and Natural Resource Management Norwegian University of Life Sciences – NMBU Ås Norway; ^4^ Departamento de Biologia Universidade Federal do Amazonas – UFAM Manaus Brazil; ^5^ Coordenação de Biodiversidade, Programa de Coleções Científicas Biológicas Instituto Nacional de Pesquisas da Amazônia – INPA Manaus Brazil

**Keywords:** beta‐diversity, community, inundation, Nymphalidae, stratification, wetlands

## Abstract

The seasonal flood pulse in Amazonia can be considered a primary driver of community structure in floodplain environments. Although this natural periodic disturbance is part of the landscape dynamics, the seasonal inundation presents a considerable challenge to organisms that inhabit floodplain forests. The present study investigated the effect of seasonal flooding on fruit‐feeding butterfly assemblages in different forest types and strata in central Amazonia. We sampled fruit‐feeding butterflies in the canopy and the understory using baited traps in adjacent upland (unflooded forests—*terra firme*), white and blackwater floodplain forests (*várzea* and *igapó*, respectively) during the low‐ and high‐water seasons. Butterfly abundance decreased in the high‐water season, especially of dominant species in *várzea*, but the number of species was similar between seasons in the three forest types. Species composition differed between strata in all forest types. However, the flood pulse only affected butterfly assemblages in *várzea* forest. The β‐diversity components also differed only in *várzea*. Species replacement (turnover) dominated the spatial β‐diversity in *igapó* and *terra firme* in both seasons and *várzea* in the high‐water season. Nonetheless, nestedness was relatively higher in *várzea* forests during the low‐water season, mainly due to the effect of dominant species. These results emphasize the importance of seasonal flooding to structure butterfly assemblages in floodplain forests and reveal the idiosyncrasy of butterfly community responses to flooding in different forest types. Our results also suggest that any major and rapid changes to the hydrological regime could severely affect floodplain communities adapted to this natural seasonal hydrological cycle, threatening the existence of these unique environments.

## Graphical abstract

This is the first study that assessed the effect of seasonal flooding pulse on butterfly communities in the Amazon. We evaluate assemblage metrics of fruit‐feeding butterflies in the low‐ and high‐water seasons in each forest type and different forest stratum. The seasonal flood increases spatial turnover in all forest types and strata.

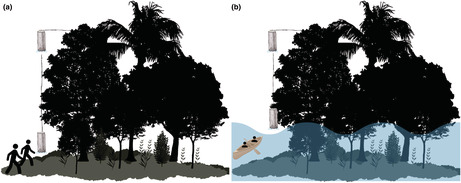

## 1 INTRODUCTION

How organisms respond to local disturbances is one of the main questions about population dynamics, resilience, and species conservation (Costa et al., 2018; Hobbs & Huenneke, 1992). Disturbance can be considered an event that changes the environment, influencing habitat dynamics and resource availability for communities and populations (Turner, 2010; White & Pickett, 1985). These can be stochastic or periodic with different drivers, intensities, and duration (Foster et al., 1998; Junk et al., 1989; Turner, 2010), and different organisms can respond differently to the same disturbance (Filgueiras et al., 2021; McKinney & Lockwood, 1999). Natural stochastic disturbances, such as tornadoes, tsunamis, earthquakes, volcanic eruptions, and even a simple tree fall resulting in a small open patch in the forest, can affect biodiversity over local and regional scales (Brown & Hutchings, 1997; Brown, 1997; Foster et al., 1998). On the other hand, natural periodic disturbances are part of the landscape dynamics. Therefore, they may act as selection filters and shape the evolution of associated biota over longer periods and geographical scales (Adis et al., 1997; Junk et al., 1989; Simon et al., 2009).

The seasonal flood pulse can be considered a primary driver of plant and animal community structure in Amazonian floodplain environments (Junk et al., 1989). Due to local and regional variation in topography, seasonal rainfall regimes, flood duration, and depth differ across the basin (Junk et al., 2015). *Várzea* forests are periodically flooded by water carrying large amounts of suspended, nutrient‐rich sediments (white water), making this forest very productive (Prance, 1979). Contrastingly, the *igapó* forest is inundated by nutrient‐poor black‐ or clear‐water rivers and is therefore less productive and contains fewer species than *várzea* (Junk et al., 2015; Pereira et al., 2009; Prance, 1979). Thus, local organisms experience different levels of flooding and heterogeneous water characteristics, which may affect their response to high‐ or low‐water seasons (Junk et al., 1989; Wittmann et al., 2004). In general, the high‐water season provides greater habitat availability, more resources, and better connectivity among floodplain features for aquatic organisms (Junk et al., 1989). However, the seasonal inundation may limit the occurrence or persistence of terrestrial species (Adis & Junk, 2002; Haugaasen & Peres, 2007). For such species to survive in these environments during the high‐water season, strategies must deal with the decreased amount of habitat and resource availability in the understory (Ramalho et al., 2021).

Several survival strategies and adaptations for dealing with seasonal floods in floodplain forests have been reported for animal taxa (Adis, 1982; Adis et al., 1997; Adis & Junk, 2002; Adis & Messner, 1997; Adis & Pagés, 2001; Junk et al., 1989; Ramalho et al., 2018, 2021). Temporary migrations to unflooded forests (horizontal migration) and vertical migrations to the midstory and canopy (Adis, 1982; Adis & Sturm, 1987; Irmler, 1979; Ramalho et al., 2021) are two alternatives for species that can displace easily (Alvarenga et al., 2018; Costa et al., 2018). Other adaptations, such as dormancy during egg or adult stages and natural or self‐made shelters, have been reported for more sedentary terrestrial animals (Adis & Junk, 2002; Adis & Sturm, 1987). Assemblage‐level impacts of flooding have also been detected. For example, there is a clear vertical stratification in pseudoscorpions species richness and ant abundance in flooded forests. Species sampled in the flooded forests were largely arboreal, whereas *terra firme* species were more abundant on the ground (Adis & Mahnert, 1990; Pringle et al., 2019).

Although the effects of seasonal flooding have been investigated for several taxa (plants [Junk, 1989; Prance, 1979; Wittmann et al., 2010]; some invertebrates [Adis & Junk, 2002; Adis & Sturm, 1987; Pringle et al., 2019]; terrestrial vertebrates [Costa et al., 2018; Haugaasen & Peres, 2007; Ramalho et al., 2021]; birds [Beja et al., 2009; Rowedder et al., 2021]; bats [Bobrowiec et al., 2014; Pereira et al., 2010]; primates [Haugaasen & Peres, 2005a]), we know surprisingly little about the impact of seasonal floods on the Amazonian floodplain butterfly fauna. Butterflies are probably the best‐known insect group with a relatively robust taxonomic resolution (Bonebrake et al., 2010). They are considered good indicators of environmental change due to their short life cycles, sensitivity to disturbances, and rapid changes to assemblage composition (Brown & Freitas, 2000; Brown, 1997). In addition, many Amazonian butterfly species portray a marked association with a particular forest type (Graça, Pequeno, Franklin, Souza, & Morais, 2017; Oliveira et al., 2021; Rabelo et al., 2021), so it is expected that any changes in the assemblage structure between seasons would be easily detected.

Butterflies also show clear forest stratification patterns (Araujo et al., 2020; DeVries, 1988; DeVries & Walla, 2001; Fordyce & DeVries, 2016; Freire et al., 2021; Graça, Pequeno, Franklin, & Morais, 2017; Lilleengen, 2016; Ribeiro & Freitas, 2012; Santos et al., 2017; Schulze et al., 2001), which can be explained by the different environmental conditions in the understory and canopy. The contrasting conditions between strata in tropical forests seem to act as an evolutionary force, as marked phylogenetic and trait signals are reported in butterflies occupying different strata (Fordyce & DeVries, 2016; Graça, Pequeno, Franklin, & Morais, 2017; Le Roy et al., 2021; Mena et al., 2020; Santos et al., 2017). In floodplain forests, understory butterflies have to deal with habitat and resource decrease and find some strategy that allows them to survive the seasonal flood.

Assessing the variation of species composition between sampling sites over time (spatiotemporal β‐diversity) can be helpful to understand how Amazonian butterfly assemblages are affected by periodic flooding. Partitioning β‐diversity into turnover and nestedness components may elucidate the main underlying process responsible for the assemblage structure (Baselga, 2010). While turnover relates to species replacement, nestedness reflects a process of species loss, where sites with a smaller number of species are a subset of richer sites. Butterfly communities tend to present high turnover values between forest types (Graça, Pequeno, Franklin, Souza, & Morais, 2017; Rabelo et al., 2021) and between seasons (Santos et al., 2017) since they are habitat‐specific and seasonal organisms (Brito et al., 2014; Emmel & Leck, 1970). However, there is no information about the spatiotemporal β‐diversity of butterfly communities related to Amazonian seasonal floods.

Here, we take advantage of a region in central Amazonia where adjacent *várzea*, *igapó*, and *terra firme* (unflooded) forest can be found to investigate how fruit‐feeding butterfly assemblages respond to seasonal inundation. We hypothesize that fruit‐feeding butterfly abundance and richness are lower during the high‐water than the low‐water season in floodplain forests (*várzea* and *igapó*), but remain stable in *terra firme* forests that do not experience flooding. We also hypothesize that fruit‐feeding butterfly composition differs between seasons and forest strata, but the former pattern will not be detected in *terra firme* forests. We further expect that the butterfly assemblage in floodplain forests has higher spatial turnover during the low‐water season due to more space and resource availability and a nestedness pattern in the high‐water season. These selective pressures imposed by flooding will be more prominent in the assemblages from the understory than in the canopy in the floodplain forests, with no difference in *terra firme*. Understanding how these key organisms respond to the flood pulse is essential given that extreme climatic events are becoming more frequent, making seasonal variations increasingly unpredictable (Barichivich et al., 2018).

## 2 MATERIALS AND METHODS

### 2.1 Study area

**FIGURE 1:**
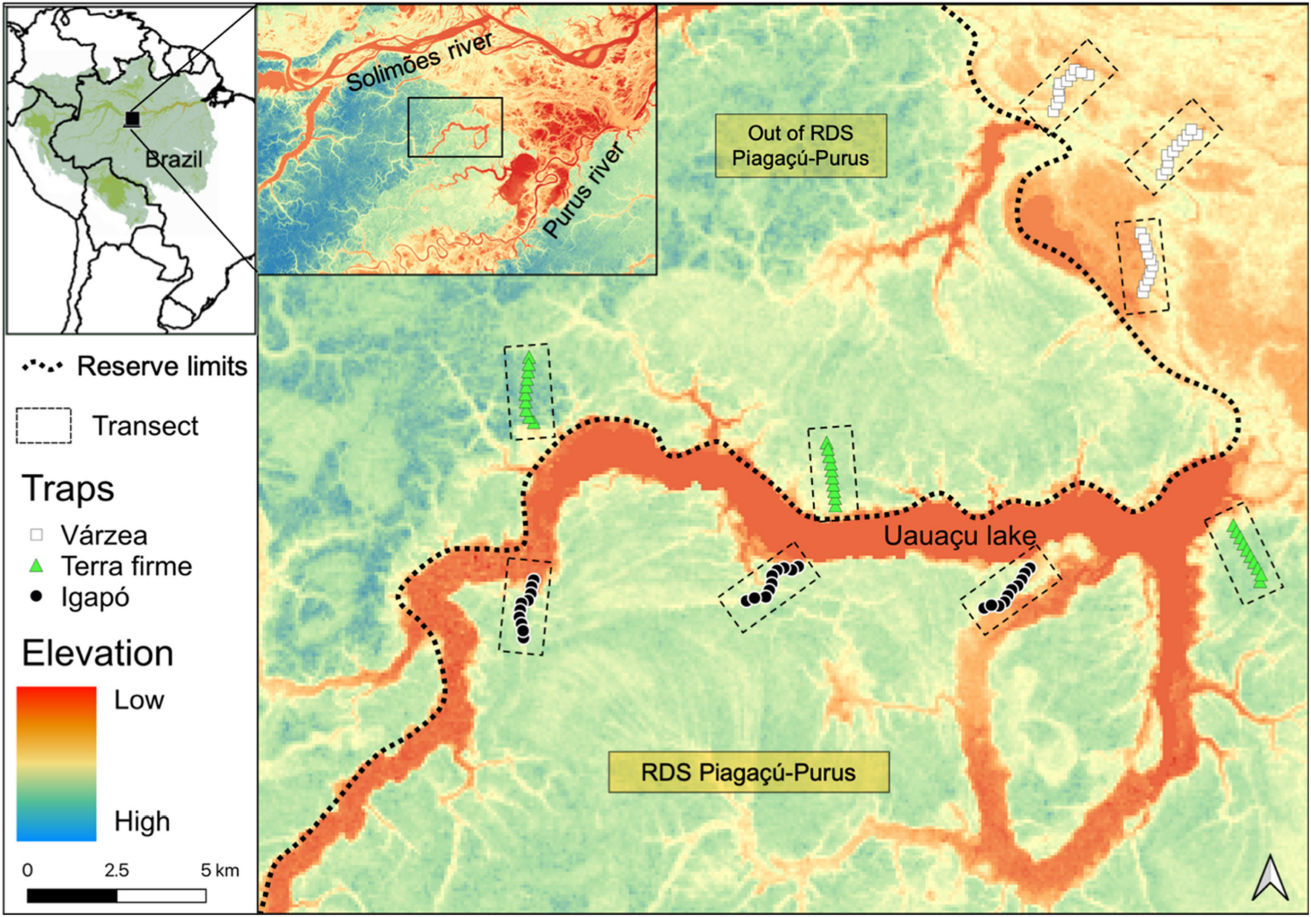
Location of the study and sampling transects (dashed rectangles) in the Uauaçu Lake region at the Piagaçú‐Purus Sustainable Development Reserve (dotted line), Amazonas, Brazil. Green triangles = *terra firme* traps, white squares = *várzea* traps, and black circles = *igapó* traps.

The study was carried out from October to November 2018 (low‐water season) and from May to June 2019 (high‐water season) around the Uauaçu Lake (4°14′S; 62°17′W) in central Amazonia. The study area is squeezed between the confluence of the Solimões and Purus rivers and most sampling sites lie within the Piagaçú‐Purus Sustainable Development Reserve (Figure 1). *Terra firme* is the dominant forest type and remains unflooded all year. The seasonally flooded forests, *várzea* and *igapó*, experience inundation for up to 6 months each year. There are different types of *várzea* forests (Wittmann et al., 2004), and in this study, we only sampled the *high*‐*várzea*, which is topographically more elevated and is flooded up to about three meters (Wittmann et al., 2004). The *igapó* forest has a shorter stature than *várzea*, and flooding reaches an average of six meters in depth.

### 2.2 Sampling design

**FIGURE 2:**
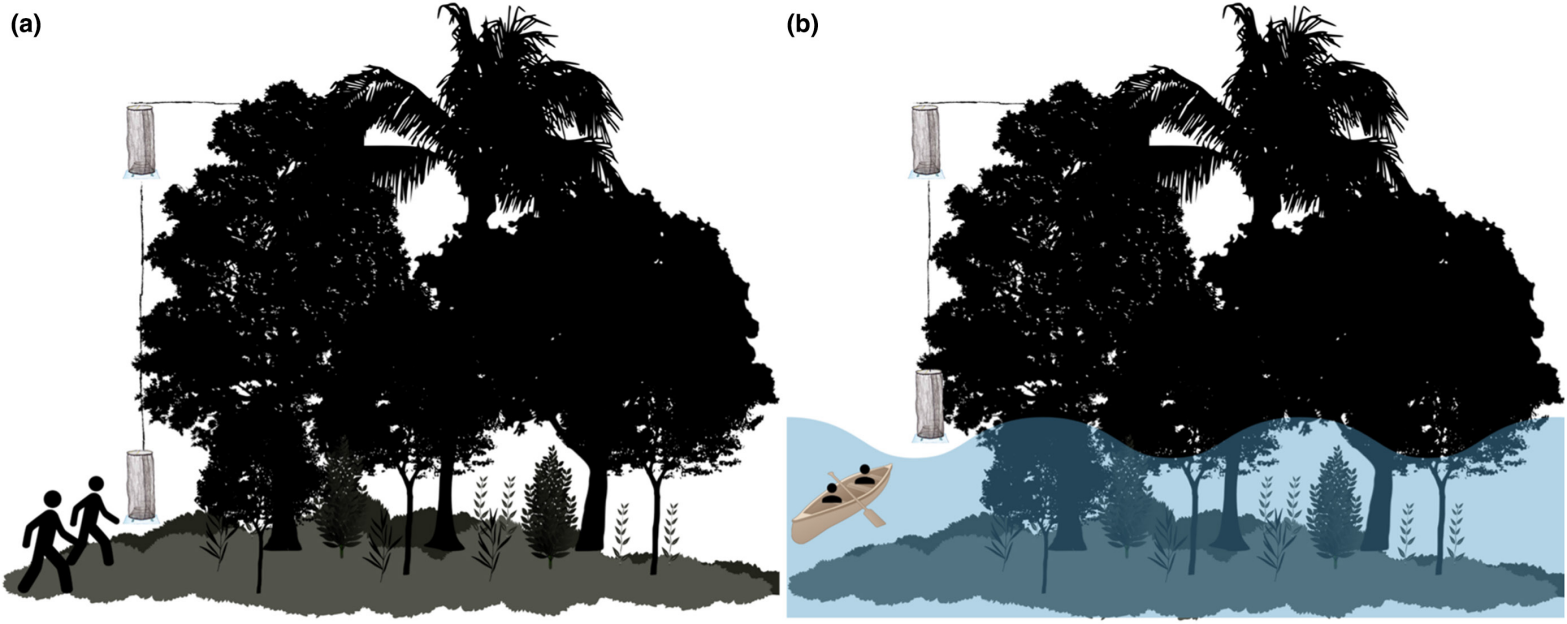
Fruit‐feeding butterfly sampling scheme in (a) low‐ and (b) high‐water seasons. Traps were placed in the canopy and understory. Trap inspection during the high‐water season in flooded forests was performed using a canoe.

We established three 2‐km transects in each forest type (*terra firme*, *igapó*, and *várzea*; Figure 1). We sampled the same transects in both the low‐ and high‐water season. In each transect, we placed 20 (10 in the understory and 10 in the canopy) cylindrical Van Someren‐Rydon‐type traps (Rydon, 1964). These were placed 200 m apart and were baited with fermented banana and brown sugar. Traps were kept open for 4 days, totaling 1440 trap/days (480 in each forest type), and bait was replaced every 48 h (Freitas et al., 2014). Butterfly trap inspection in floodplain forests was performed using a canoe during the high‐water season (Figure 2). During this sampling period, understory traps were placed immediately (0.5–1 m) above the water surface, varying in the forest stratum height, depending on the topographic elevation.

Butterflies were identified to species or subspecies using guides (e.g., www.butterfliesofamerica.com; D'Abrera, 1987a, 1987b) and taxonomic literature (Penz et al., 2014; Zacca et al., 2021). An experienced butterfly taxonomist (T. Zacca) also verified all species identifications. All collected butterflies were deposited at the Entomological Collection of the National Institute of Amazonian Research (INPA). Some individuals of the most abundant species were also deposited at the Community Ecology Lab at the Federal University of Amazonas (UFAM).

### 2.3 Statistical analysis

Species richness for each season and forest type was evaluated using rarefaction curves indicating interpolated and extrapolated values based on number of individuals. Additionally, we ran a sample‐based analysis to assess the species richness controlled by species frequency in sampling units in each season. This method minimizes the abundance effect on species diversity. For this analysis, we used each combined trap (understory and canopy) as a sampling unit (30 sampling units per season and per habitat). We also ran coverage‐based rarefaction and extrapolation curves for each forest type, which indicate how the sampling completeness varies with the number of individuals sampled in each season (Chao et al., 2014). Rarefactions and extrapolation curves were based on Hill numbers via three functions: qD = 0 (Richness), qD = 1 (Shannon), and qD = 2 (Simpson) generated in the “iNEXT” package in R (Chao et al., 2014; Colwell et al., 2012; Hsieh et al., 2016). The result of Shannon and Simpson curves can be found in Figure S1.

To check whether the butterfly species composition of each forest type differs among seasons and whether the composition in each forest stratum is affected by seasonality, we performed a PERMANOVA (Permutational Multivariate Analysis of Variance), with season (high‐ and low‐water) and stratum (understory and canopy) as independent variables in each forest type separately. In this case, the sampling unit was each transect (10 combined traps) per season and stratum. Although PERMANOVA is a highly used method, in some cases it may confound location with dispersion effects. For example, differences may be detected by within‐group variation (dispersion) instead of different mean values of the groups (Warton et al., 2012). Therefore, we also ran a multivariate homogeneity of group dispersions (Anderson, 2006) to check whether differences detected by PERMANOVA were from differences in point location (composition). Multivariate homogeneity of group dispersions (Betadisper) is a multivariate analogue of Levene's test for homogeneity of variances and was based on spatial median in our case. In both analyses, we used abundance data with Bray–Curtis dissimilarity measures. P‐values were calculated based on 999 permutations.

β‐diversity (the variation in species composition between two assemblages—in time or space) can be measured in different ways (Tuomisto, 2010a, 2010b). We followed the Baselga framework (Baselga, 2010), adapted by Legendre (2014), to work with abundance data. In this approach, the β‐diversity is partitioned into turnover and nestedness components. The turnover measures the ratio at which one set of species replaces another between locations or seasons, reflecting the influence of local variables on community structure. This is related to the degree of ecological tolerance (or specificity) of the species and the breadth of their niches (Legendre, 2014). The nestedness measures the difference in species richness between sites or seasons, reflecting species loss. It is defined by the degree of dissimilarity between areas or periods, but only in the form of subsets of species, where sites or seasons with less species richness are compositional subsets of a more diverse location/season (Baselga, 2010; Legendre, 2014; Podani & Schmera, 2011).

We assessed the spatial variation of the fruit‐feeding butterfly species composition (β‐diversity) using Sørensen dissimilarity (Baselga, 2010, 2013; Legendre, 2014). We used abundance data of combined traps (understory and canopy) to estimate which component (turnover or nestedness) predominated the fruit‐feeding butterfly assemblage structure in each season (low‐ and high‐water seasons) of each forest type (Legendre, 2014). To assess the spatial β‐diversity, we obtained a multisite dissimilarity value for each β‐diversity components in each forest type and season separately using the “*beta_div*” function (Legendre, 2014). We opted for multisite comparison, because pairwise dissimilarity tends to have a large variation and in some cases may not properly quantify multiple‐site compositional heterogeneity (Baselga, 2013). We ran the same analysis for each stratum (understory and canopy) separately in each forest type to assess β‐diversity components values but using each trap as sampling unit. To assess whether multisite β‐diversity components differ among season, forest type, and stratum, we used bootstraps to estimate means and standard deviations of each component. We identified significant differences by the lack of overlap between 95% confidence intervals, estimated here as 2* the standard deviation (Manly, 2007). All bootstrap analyses were based on 999 randomizations using half of the samples in each permutation. All analyses were performed on R, version 4.1.0 (R Core Team, 2021).

## 3 RESULTS

### 3.1 Low‐ and high‐water season butterfly assemblages

We sampled 285 individuals of 58 species of fruit‐feeding butterflies (Table S1). A total of 42 species (199 individuals) were sampled in the low‐water season and 39 species (86 individuals) in the high‐water season. *Terra firme* had slightly more species and individuals in the high‐water season. *Várzea* had the same species richness in both seasons, but with fewer individuals in the high‐water season. *Igapó* had lower species richness and abundance in the high‐water season (Table 1).

**TABLE 1:** Species richness and total abundance of fruit‐feeding butterflies sampled in each flooding season and forest type in Uauaçu lake region, Amazonas, Brazil.

Forest type	Low‐water	High‐water	Both seasons
Terra firme	13 (*N* = 21)	17 (*N* = 28)	7 (*N* = 29)
Várzea	19 (*N* = 130)	19 (*N* = 41)	11 (*N* = 125)
Igapó	24 (*N* = 48)	12 (*N* = 17)	8 (*N* = 34)

We had a low capture rate in the traps. In the *terra firme*, 24% of traps had at least one individual in the low‐water season and 29% in the high‐water season. In *várzea*, 60% of the traps in the low‐water season had captures and in the high‐water season this rate decreased to 40%. In *igapó*, 42% of traps had captures in the low‐water season, and 27% in the high‐water season.

Singletons and doubletons were represented by 30 species (51.7% of total species). In the low‐water season, singletons and doubletons comprised 52.3% of species, while in the high‐water season, this rate increased to 66.6%. The six most abundant species in the low‐water season were *Taygetis mermeria* (*n* = 38), *Chloreuptychia herseis* (*n* = 21), *Pseudodebis valentina* (*n* = 15), *Pseudodebis marpessa* (*n* = 10), *Magneuptychia ocnus* (*n* = 10), and *Magneuptychia aff ocnus* (*n* = 10), together accounting for 52.2% of individuals captured in this season. In the high‐water season, *Pseudodebis valentina* (*n* = 6), *Pseudodebis marpessa* (*n* = 6), *Bia actorion* (*n* = 5), *Chloreuptychia herseis* (*n* = 5), *Temenis laothoe* (*n* = 5), and *Catonephele acontius* (*n* = 5) were the six most abundant species, representing 37.2% of individuals.

### 3.2 Fruit‐feeding butterfly richness and abundance

**FIGURE 3:**
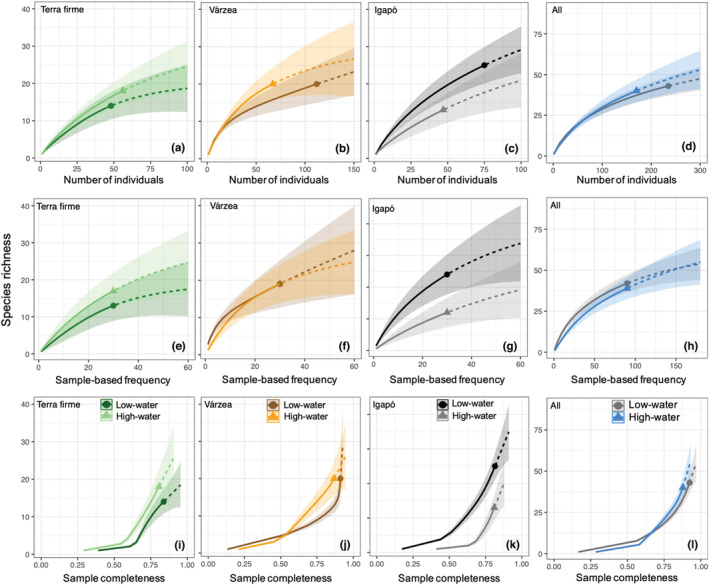
Individual‐based rarefaction (a–d), sample‐based rarefaction (e–h), and sample completeness (i–l) curves for low‐ and high‐water seasons (circles and triangles, respectively) of fruit‐feeding butterfly assemblages in each individual forest type and combined (all). Solid lines represent the interpolated and dashed lines the extrapolated values. Shaded areas represent 95% confidence intervals.

**FIGURE 4:**
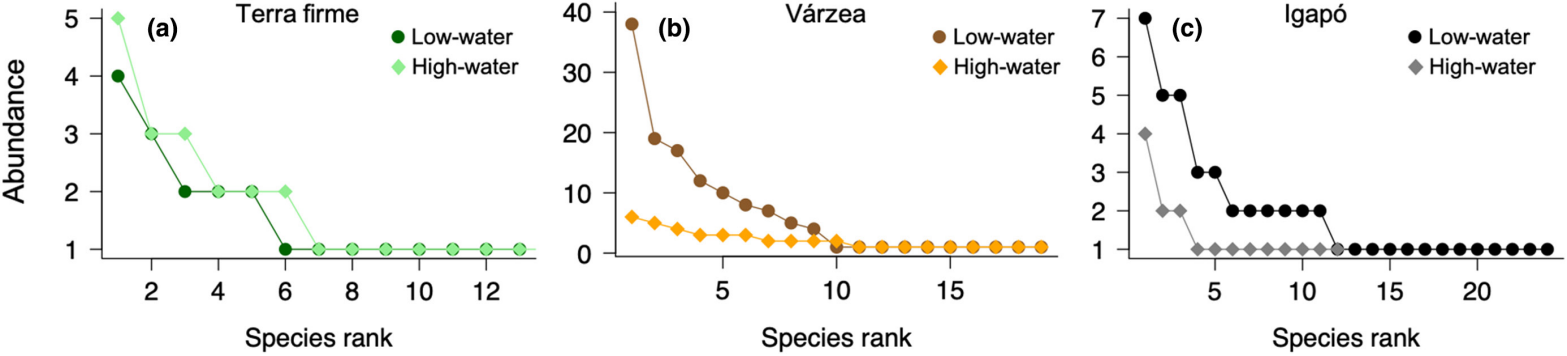
Species‐rank abundance distribution in low‐ (circles) and high‐water (diamonds) seasons in each forest type; (a) *terra firme*, (b) *várzea*, and (c) *igapó*. Note the high number of species represented by a single individual in *várzea* during the low‐water season.

Overall, the number of species sampled in the high‐water season was similar to that in the low‐water season, even though fewer individuals were sampled in the high‐water season (Figure 3d). According to the individual‐based rarefaction analysis, the curves of *terra firme* and *várzea* suggest that the high‐water season supports more species than the low‐water season, but a contrasting result was found in *igapó* (Figure 3a–d). However, the curves showed a large confidence interval overlap, especially in each habitat separately. This pattern emerged largely because three species (e.g., *Taygetis mermeria* and *Chloreuptychia herseis* in *várzea*, and *Chloreuptychia chlorimene* in *igapó*) dominated the sample in the low‐water season, as shown by the species‐rank abundance distribution (Figure 4). Nonetheless, when the abundance effect is controlled by the species frequency on sampling units, *terra firme* and *várzea* assemblages did not show differences in the rarefaction curves between seasons (Figure 3e,f). On the contrary, the assemblage from *igapó* in the low‐water season is expected to support more species (Figure 3g). Sample completeness was therefore higher during the low‐water season in all forest types (Figure 3i–l).

### 3.3 Fruit‐feeding butterfly composition across seasons and forest strata

Species composition between the canopy and understory differed in all forest types (Table 2). However, only in *várzea* did the composition differ between strata across the two seasons (Table 2). We did not detect differences in dispersion between strata and season among the forest types (Table S2), suggesting that differences between strata for all forest types and between season in *várzea* forests are due to differences in species composition, rather than sampling heterogeneity between groups.

**TABLE 2:** Results of the compositional analysis (PERMANOVA) using “stratum” and “season” as variables.

	Terra firme			Várzea			Igapó		
	*F*	*R* ^2^	*p*	*F*	*R* ^2^	*p*	*F*	*R* ^2^	*p*
Stratum	1.99	.18	.020*	4.01	.29	.001***	3.47	.26	.001***
Season	0.64	.06	.771	2.69	.19	.005**	1.47	.11	.181

*Note*: Asterisks highlight significant differences (*p ≤ .05, **≤.01, ***.001) in fruit‐feeding butterfly composition.

### 3.4 Spatial β‐diversity of fruit‐feeding butterflies per season

**FIGURE 5:**
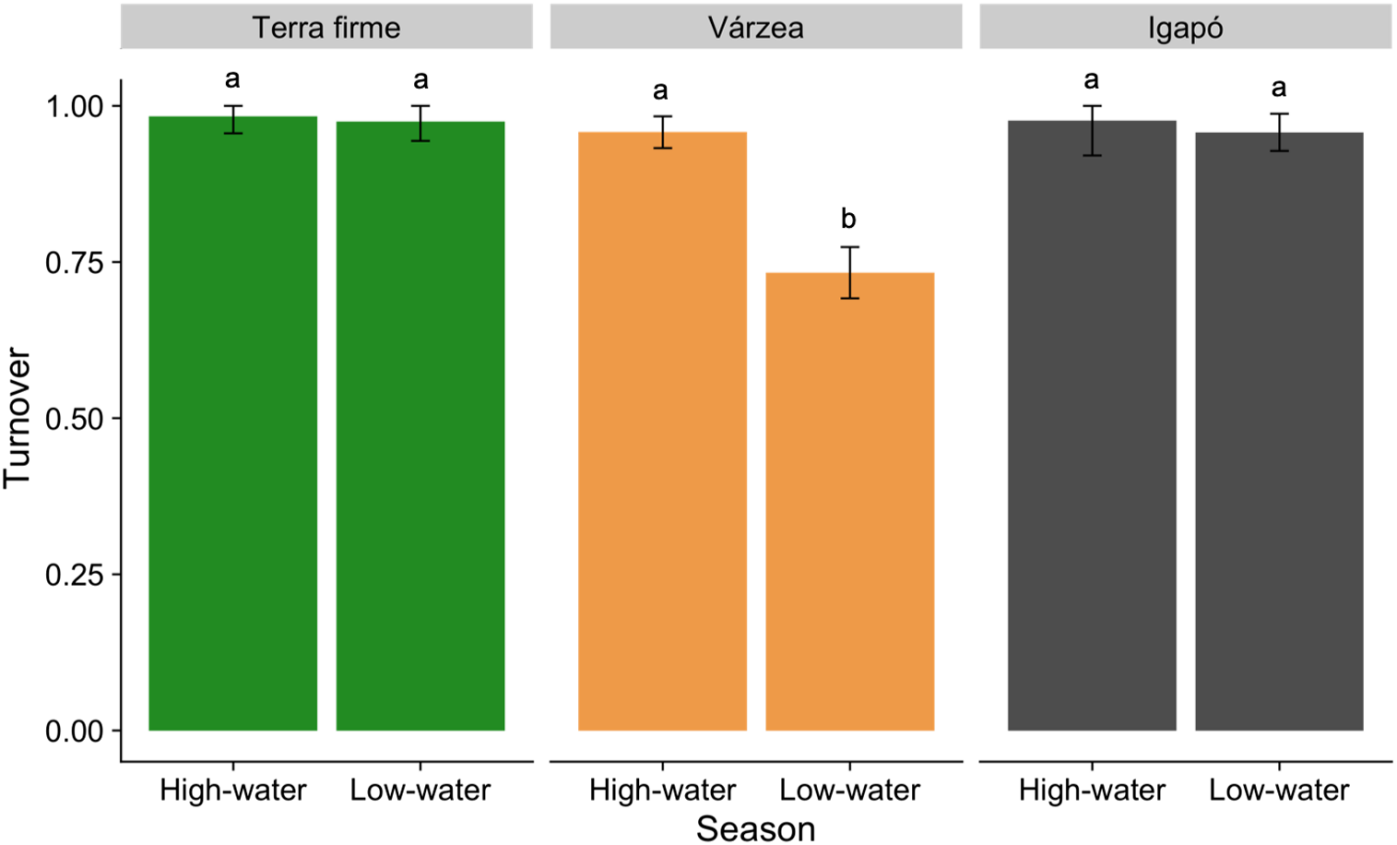
Relative contribution of spatial turnover among the forest types per season at the Uauaçu Lake, central Amazonia, Brazil. The bars represent the observed multisite component values, and the black lines represent 95% confidence intervals. The values are statistically different between seasons where the 95% confidence intervals do not overlap. Different letters indicate significant differences in turnover values between seasons.

**FIGURE 6:**
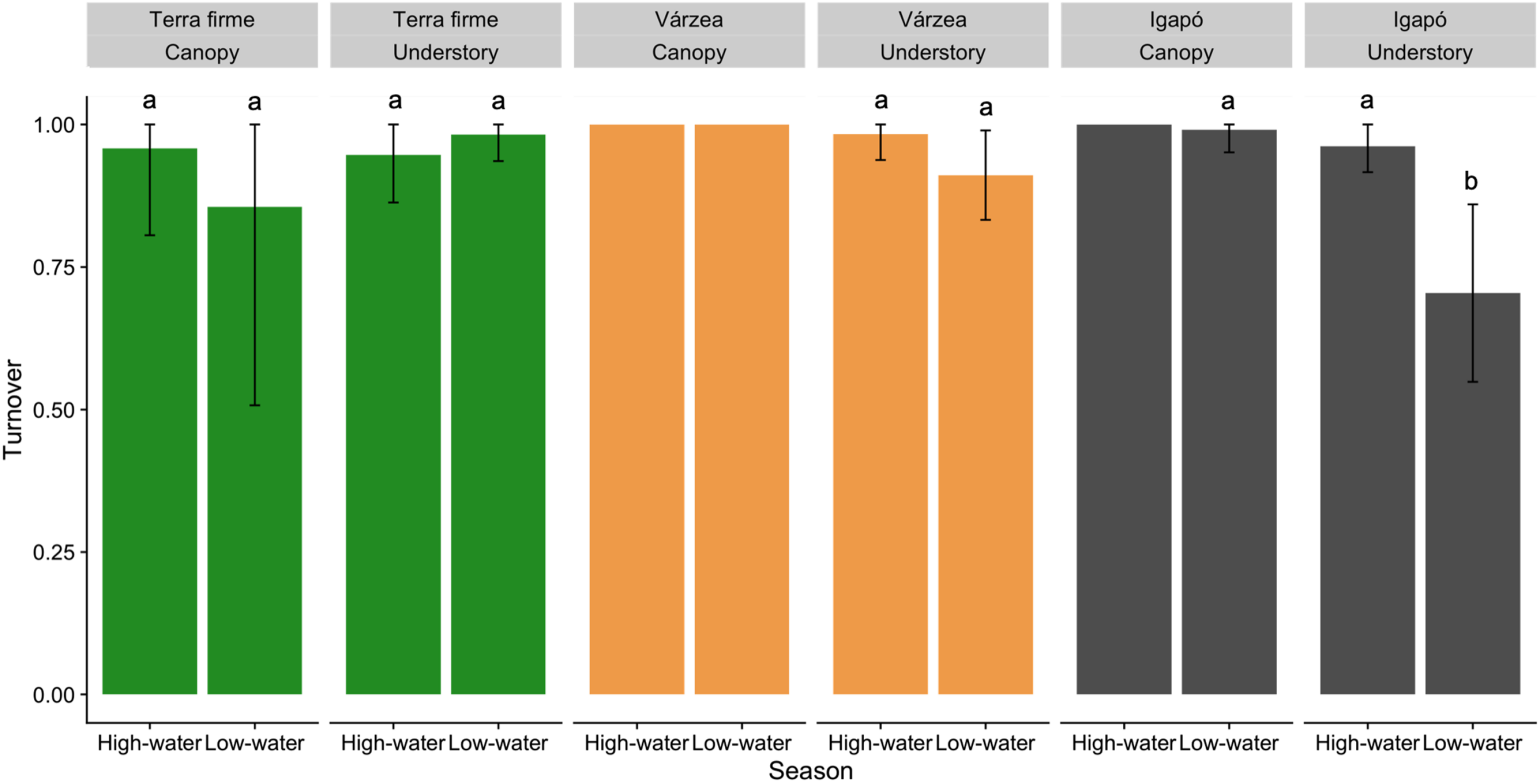
Butterfly species turnover values with abundance data for each stratum per season in *terra firme* (green), *várzea* (orange), and *igapó* (black). The black bars represent 95% confidence intervals based on 999 bootstraps randomization. The turnover dominated the canopy of *várzea* and canopy of *igapó* in the high‐water season, leading to no variation in bootstrap randomizations. Different letters indicate significant differences in turnover values between seasons and strata.

Species turnover dominated the butterfly β‐diversity in *terra firme* and *igapó* in both seasons and in *várzea* during the high‐water season (Figure 5). Since turnover and nestedness are complementary, the higher turnover in *várzea* in the high‐water season means that nestedness had a relatively higher contribution in the low than high‐water season. Among strata, assemblages from *terra firme* and *várzea* did not show differences in species turnover values. On the other hand, *igapó* assemblages presented differences in species turnover between strata in the low‐water season, and in the understory between seasons (Figure 6).

## 4 DISCUSSION

The disturbance caused by flooding in Amazonian floodplain forests and its influence on species and community dynamics has been studied for decades (Adis, 1977; Beck, 1969). Nonetheless, this is the first study to investigate the effect of the flood pulse on Amazonian butterfly assemblages. We found that the fruit‐feeding butterfly assemblage in the two floodplain forest types responded differently to flooding. *Várzea* presented differences in the butterfly assemblage structure between the low‐ and high‐water seasons, and this difference was mainly due to a considerable loss of butterfly individuals during the inundation period. The butterfly assemblage in *igapó* did not show significant structural differences between low‐ and high‐water seasons, despite a decrease in abundance. As expected, the butterfly assemblage from *terra firme* did not show significant assemblage structure changes between seasons.

We expected that the understory species would disappear in both floodplain forest types due to the seasonal inundation, increasing the number of species and individuals in the adjacent *terra firme* during the high‐water peak through horizontal migration. We found that the flooding dramatically impacted butterfly density in floodplain forests, decreasing the number of individuals, but surprisingly, butterfly richness remained stable between seasons. This pattern was found because we captured more dominant species in these forests during the low‐water season, particularly in *várzea*. The number of singletons and doubletons was high in both seasons, but this is in line with previous fruit‐feeding butterfly inventories in central Amazonia (Graça, Souza, Franklin, Morais, & Pequeno, 2017; Rabelo et al., 2021; Spaniol et al., 2019). The lower butterfly abundance during the high‐water season in floodplain forests may be a consequence of the lack of host plants for immature oviposition and feeding in the understory, as the most abundant species in the low‐water season were monocot feeders. In addition, many floodplain trees shed their leaves during the inundation phase in the study area (Haugaasen & Peres, 2005b).

There are no studies investigating adult butterfly resource use in flooded forests. However, although the habitat of understory floodplain butterflies is completely flooded in the high‐water season (especially in *igapó*), there are mechanisms in which their persistence in floodplain forest during the flood can be maintained. The high‐water period is the main fruiting season of floodplain trees (Haugaasen & Peres, 2005b), as many trees are water‐ or fish‐dispersed (Kubitzki & Ziburski, 1994). Therefore, there are some resources available in the forest, from which fruit‐feeding butterflies can obtain their nutritional requirements. Schulze et al. (2001) suggested that epiphytes could act as traps for falling fruit and in this way provide a source of rotting fruit in the midstory immediately above the floodwater. The same goes for animal feces and carcasses that some of these butterflies may also feed on (Freitas et al., 2014). Since epiphytes are known to be abundant in *várzea* (Leimbeck & Balslev, 2001), this could be one explanation of how the remaining understory individuals are able to persist above the water in floodplain forests during the flooding.

We notice that the abundance of butterflies captured is lower in our study compared with other studies of fruit‐feeding butterflies in central Amazonia (Graça, Souza, Franklin, Morais, & Pequeno, 2017; Rabelo et al., 2021; Ribeiro & Freitas, 2012; Spaniol et al., 2019). However, some of these studies used entomological net samples to complement the baited trap sampling. We decided to not use entomological net samples because we wanted to assure an equal sampling effort in the canopy and understory, and hand‐net sampling would inflate understory records (Fordyce & DeVries, 2016). Also, none of these studies were done in the high‐water season in flooded forests. We therefore attribute the overall lower abundance to the seasonal inundation. However, *terra firme* does not experience flooding. The low capture success in this forest type could reflect a very low population size of the different species, but the reasons why this region supports such a low density of fruit‐feeding butterflies still need further investigations and long‐term studies. Perhaps the low population density can be explained by the influence of a vast floodplain area in the region, or the fact that *terra firme* transects were close to the lake margin and not in core *terra firme* areas that are logistically difficult to visit. Rabelo et al. (2021) also found less species of Nymphalidae in *terra firme* compared with *várzea*, and the *terra firme* forest of that study is also bordered by a vast floodplain.

In *terra firme*, there was a slightly higher number of individuals and species of fruit‐feeding butterflies in the high‐water season compared with the low‐water season. As we did not track individuals, we are unable to assess whether the new species records at this time are due to horizontal migration of butterflies seeking to escape the floodplains during flooding. However, we note that none of these species (*Amiga arnaca*, *Catoblepia berencynthia*, *Catoblepia soranus*, *Chloreuptychia rectilinea*, *Cithaerias aurora*, *Eunica eurota*, *Magneuptychia fugitiva*, and *Tigridia acesta*) were sampled in floodplain forests during the low‐ or high‐water season (Figure S2). As the butterfly species tend to be very habitat‐specific (Oliveira et al., 2021), and some of them persist for long periods in the same home range (Brown & Hutchings, 1997), it is likely that these species were not sampled in the low‐water season in *terra firme* due to detection constraints caused by low abundances or the temporal variation in their life stages (DeVries & Walla, 2001; Ribeiro et al., 2016). In addition, some of these species are vulnerable to disturbances due to poor dispersal capacity (Spaniol et al., 2019) and are therefore probably not found in floodplain forests.

Our results show that butterfly assemblage composition changed between seasons only in *várzea*, revealing that assemblages from floodplains further from *terra firme* respond differently to those close to *terra firme*. *Igapó* presented a similar butterfly composition between seasons. This pattern can be explained by the presence of strong flyers (e.g., Biblidinae, Charaxinae, and Nymphalinae subfamilies) and, as *igapó* is intertwined with *terra firme* in this landscape, the individuals may use *terra firme* and its borders as a refuge during flooding (Beja et al., 2010). The compositional changes in *várzea* were mainly due to the loss of individuals from the dominant butterfly species in the high‐water season. Although the most abundant butterflies in *várzea* are not exclusively found in this forest type (Emmel & Austin, 1990), they seem to prefer this habitat. Most of them are part of the Satyrini tribe, in which the larvae feed on grasses and bamboos (Beccaloni et al., 2008; DeVries, 1985) that are abundant in these highly productive floodplains during the low‐water season (Brown & Hutchings, 1997; Oliveira et al., 2021; Rabelo et al., 2021). This butterfly group has a low dispersal capacity (Fordyce & DeVries, 2016), and needs to deal with habitat decrease during the flood period. However, we note that *várzea* sites contained more fast‐flying butterflies during the high‐water season than in the low‐water season (e.g., *Hamadryas* ssp., *Historis odius*).

The species composition differed between the understory and canopy in all forest types. This is a common pattern found in tropical forest butterfly communities where many species show a strong association with a particular forest stratum (Araujo et al., 2020; DeVries, 1988; Fordyce & DeVries, 2016; Freire et al., 2021; Graça, Pequeno, Franklin, & Morais, 2017; Ribeiro & Freitas, 2012; Santos et al., 2017; Schulze et al., 2001). However, the assemblage composition within each stratum only changed between seasons in *várzea*. Fruit‐feeding butterflies inhabiting the floodplain understory are directly affected by flooding as the inundation covers their understory habitat and decreases niche availability. The effect of flooding on the understory butterfly assemblage in *várzea* is more significant than in *igapó—*shown by the larger decrease in number of individuals in *várzea* during this period. The high‐water season in *várzea* presents a peak of mature fruits (Haugaasen & Peres, 2005b), which may attract strong and fast‐flying individuals usually found in the forest canopy (DeVries, 1988; DeVries & Walla, 2001). Although some organisms migrate vertically to escape the flooding (Adis, 1982; Adis & Sturm, 1987; Irmler, 1979; Ramalho et al., 2021), we show that butterflies follow the water level, but do not change their stratum affiliation. Thus, the difference in composition between seasons and strata in *várzea* is possibly due to species replacement caused by the dramatic decrease in abundance of dominant species in the understory and the appearance of fast‐flying butterflies, likely due to fruitification in the canopy.

The β‐diversity components between seasons also differed only in *várzea*. Species turnover dominated the spatial β‐diversity in *igapó* and *terra firme* in both seasons. On the contrary, the nestedness component in *várzea* during the low‐water season was proportionally higher than in the high‐water season, mainly due to the effect of dominant species in the understory. A higher turnover in *terra firme* in both seasons was expected, especially in the high‐water season, due to the possibility of individuals arriving from the flooded forests (Haugaasen & Peres, 2005a). However, the results found in the floodplains reject our hypothesis that assemblages would be structured by nestedness in the high‐water season. The same process may explain the higher turnover in *igapó*. Butterflies from *igapó* may use adjacent *terra firme* borders as refuge, especially in the high‐water season (Beja et al., 2010; Oliveira et al., 2021). In *várzea*, this result may reflect the effect of environmental disturbance caused by flooding and movements caused by fruit production (Pereira et al., 2010).

The seasonal flood did not influence species turnover in any particular stratum in *terra firme* and *várzea*, however, increased species turnover in the understory of *igapó*. We expected a more nested pattern, since *igapó* experiences a more intense flood due to its low topography and forest stratum height. In fact, although the understory assemblages from *várzea* had the largest reduction in butterfly abundance, a reasonable part of the mid‐understory is still available. On the contrary, species found in the understory of *igapó* have to deal with less available habitat and may count on greater flight ability. Therefore, this contrasting result of flood effect in the understory of different floodplain types reflects differences in butterfly species traits (Oliveira et al., 2021). This can be related to the intensity of the flood, amount of available habitat, vegetation structure, and proximity of *terra firme*.

The seasonal flooding acts as an environmental filter for floodplain forest inhabitants, and the selective pressures caused by this ancient process shape the community according to these environmental conditions (Junk, 1989). Our rapid surveys suggest that seasonal flooding plays an essential role in structuring butterfly communities on the floodplains, and the responses of the communities to the flood can vary among forest types. Further work is needed to assess how floodplain forest butterfly assemblages respond to flooding in the long‐term, and whether the different responses to floods in *igapó* and *várzea* are due to proximity to *terra firme* or due to structural differences of the floodplain forests. This is important as climate change and hydropower installations result in more unpredictable and extreme changes in water levels that may dramatically affect floodplain forests (Barichivich et al., 2018; Schöngart et al., 2021). Any major and rapid modification in the hydrological regime is therefore likely to severely affect local communities on the floodplains that are adapted to this natural seasonal hydrological cycle (Wittmann et al., 2010, 2013)—threatening the existence of these unique environments and the high number of species restricted to or associated with them (Junk et al., 2020; Laranjeiras et al., 2019).

## AUTHOR CONTRIBUTIONS

**Isabela Freitas Oliveira:** Conceptualization (equal); data curation (lead); formal analysis (lead); investigation (lead); methodology (equal); project administration (equal); writing – original draft (lead); writing – review and editing (lead). **Fabricio Beggiato Baccaro:** Conceptualization (equal); formal analysis (equal); funding acquisition (equal); investigation (equal); methodology (equal); resources (equal); supervision (equal); writing – original draft (equal); writing – review and editing (equal). **Fernanda P. Werneck:** Formal analysis (supporting); supervision (equal); writing – original draft (equal); writing – review and editing (equal). **Torbjørn Haugaasen:** Conceptualization (equal); formal analysis (supporting); funding acquisition (lead); investigation (equal); methodology (equal); project administration (lead); resources (lead); supervision (lead); writing – original draft (equal); writing – review and editing (equal).

## FUNDING INFORMATION

I.F.O. would like to thank the Coordenação de Aperfeiçoamento de Pessoal de Nível Superior—CAPES for her doctoral and doctoral sandwich fellowships (#88882.347460/2019‐01; #88881.623028/2021‐01). The study was supported by grants from the Faculty of Environmental Sciences and Natural Resource Management (NMBU) to T.H., Fundação de Amparo à Pesquisa do Estado do Amazonas—FAPEAM (N. 016/2014 PPP) and Conselho Nacional de Desenvolvimento Científico e Tecnológico—CNPq (#313986/2020‐) to F.B.B.; F.P.W. and F.B.B. would like to thank CNPq for their productivity fellowship (#311504/2020‐5 and #313986/2020‐7).

## CONFLICT OF INTEREST

The authors declare no conflict of interest.

## Supporting information


Appendix S1

## Data Availability

All data that support the findings of this study are available in the Appendix S1 of this article, and all R‐scripts and datasets are available on GitHub at https://github.com/IsaBio/Butterfly‐responses‐to‐seasonal‐flooding.git.
