# Supplementary material for: Seasonal flooding decreases fruit‐feeding butterfly species dominance and increases spatial turnover in floodplain forests of central Amazonia
Source: Ecol Evol. 2023 Jan 6;13(1):e9718. doi: 10.1002/ece3.9718 (PMC9817189; doi:10.1002/ece3.9718)
Supplement: Supplementary file 1 — Appendix S1 [file ECE3-13-e9718-s001.docx]

**SUPPORTING INFORMATION**

**Seasonal flooding decreases fruit-feeding butterflies species dominance and increases spatial turnover in the floodplain forests in central Amazonia**

I.F. Oliveira, F.B. Baccaro, F.P. Werneck, T. Haugaasen

Table S1. Fruit-feeding butterfly abundances in each stratum, in low and high-water seasons in the three forest types in Uauaçu Lake region, central Amazonia, Brazil. C= canopy, U = understory

| **Forest Type** | **Igapó** | | | | **Terra firme** | | | | **Várzea** | | | |
| --- | --- | --- | --- | --- | --- | --- | --- | --- | --- | --- | --- | --- |
| **Season** | **High** | | **Low** | | **High** | | **Low** | | **High** | | **Low** | |
| **Strata** | **C** | **U** | **C** | **U** | **C** | **U** | **C** | **U** | **C** | **U** | **C** | **U** |
| **Subfamily/ Species** |  |  |  |  |  |  |  |  |  |  |  |  |
| **Biblidinae** | **1** | **3** | **3** | **12** | **3** | **3** | **2** | **6** | **5** | **2** | **1** | **3** |
| *Catonephele acontius* |  | 2 |  | 5 | 1 | 2 | 1 | 1 |  |  |  | 1 |
| *Catonephele antinoe* |  | 1 | 1 | 4 |  |  |  |  |  | 1 |  | 1 |
| *Catonephele numilia* |  |  |  |  |  |  | 1 | 2 |  |  |  |  |
| *Eunica eurota* |  |  |  |  | 1 |  |  |  |  |  |  |  |
| *Eunica orphise* |  |  | 1 | 1 |  |  |  |  |  |  |  |  |
| *Hamadryas arinome* |  |  | 1 |  |  |  |  | 1 |  |  |  | 1 |
| *Hamadryas feronia* |  |  |  | 1 |  |  |  |  |  | 1 |  |  |
| *Hamadryas laodamia* |  |  |  |  |  |  |  |  | 2 |  |  |  |
| *Nessaea obrinus* |  |  |  | 1 |  | 1 |  | 2 |  |  |  |  |
| *Temenis laothoe* | 1 |  |  |  | 1 |  |  |  | 3 |  | 1 |  |
| **Charaxinae** | **1** | **5** | **4** | **6** | **3** | **1** | **3** | **2** | **1** |  |  |  |
| *Archaeoprepona demophon demophon* |  | 4 |  | 2 |  |  |  |  |  |  |  |  |
| *Archaeoprepona demophoon* |  | 1 |  |  |  |  |  |  |  |  |  |  |
| *Memphis laertes* |  |  |  |  |  |  | 1 |  |  |  |  |  |
| *Memphis leonida* |  |  |  |  | 2 | 1 |  | 1 |  |  |  |  |
| *Memphis moruus moruus* |  |  | 1 |  |  |  |  |  | 1 |  |  |  |
| *Memphis phantes vicina* |  |  |  | 1 |  |  |  |  |  |  |  |  |
| *Memphis polycarmes* |  |  |  | 1 |  |  |  |  |  |  |  |  |
| *Prepona dexamenus* |  |  | 2 |  |  |  |  |  |  |  |  |  |
| *Prepona laertes demodice* |  |  | 1 | 1 |  |  | 1 |  |  |  |  |  |
| *Siderone galanthis galanthis* | 1 |  |  |  |  |  |  |  |  |  |  |  |
| *Zaretis isidora* |  |  |  | 1 | 1 |  | 1 |  |  |  |  |  |
| *Zaretis itys* |  |  |  |  |  |  |  | 1 |  |  |  |  |
| **Nymphalinae** | **2** | **2** | **2** | **2** | **2** |  |  |  | **2** |  |  |  |
| *Baeotus aeilus* |  | 1 |  |  |  |  |  |  |  |  |  |  |
| *Colobura dirce* |  |  |  | 2 |  |  |  |  |  |  |  |  |
| *Historis acheronta* | 2 |  | 1 |  | 1 |  |  |  |  |  |  |  |
| *Historis odius* |  | 1 | 1 |  |  |  |  |  | 2 |  |  |  |
| *Tigridia acesta* |  |  |  |  | 1 |  |  |  |  |  |  |  |
| **Satyrinae** |  | **3** | **1** | **18** | **1** | **15** |  | **8** | **1** | **30** | **18** | **108** |
| *Amiga arnaca arnaca* |  |  |  |  |  | 1 |  |  |  |  |  |  |
| *Amphidecta calliomma* |  |  |  |  |  |  |  |  |  | 4 |  |  |
| *Amphidecta pignerator pignerator* |  |  |  |  |  |  |  |  |  | 1 |  |  |
| *Bia actorion* |  |  |  |  |  | 5 |  | 4 |  |  |  |  |
| *Cepheuptychia romani* |  |  |  |  |  |  |  |  |  |  | 1 |  |
| *Catoblepia berecynthia* |  |  |  |  | 1 | 1 |  |  |  |  |  |  |
| *Catoblepia soranus* |  |  |  |  |  | 1 |  |  |  |  |  |  |
| *Catoblepia xanthicles* |  |  |  | 1 |  |  |  |  |  |  |  |  |
| *Chloreuptychia chlorimene* |  |  |  | 7 |  |  |  |  |  |  |  |  |
| *Chloreuptychia herseis* |  | 1 |  | 3 |  | 2 |  | 1 |  | 2 |  | 17 |
| *Chloreuptychia rectilinea* |  |  |  |  |  | 2 |  |  |  |  |  |  |
| *Chloreuptychia tolumnia* |  |  |  | 1 |  |  |  |  |  |  |  | 4 |
| *Cissia myncea* |  |  |  |  |  |  |  |  |  |  | 6 | 2 |
| *Cithaerias aurora* |  |  |  |  |  | 1 |  |  |  |  |  |  |
| *Erichthodes antonina* |  |  |  | 2 |  |  |  |  |  | 1 |  | 1 |
| *Hermeuptychia maimoune* |  |  |  |  |  |  |  |  |  | 1 | 1 |  |
| *Magneuptychia aff ocnus* |  |  |  |  |  |  |  |  |  |  | 2 | 8 |
| *Magneuptychia fugitiva* |  |  |  |  |  | 1 |  |  |  |  |  |  |
| *Magneuptychia ocnus* |  |  |  |  |  |  |  |  |  | 2 | 6 | 4 |
| *Morpho achilles* |  |  |  | 1 |  |  |  |  |  |  |  |  |
| *Opsiphanes invirae* |  | 1 | 1 |  |  |  |  |  |  |  |  |  |
| *Opsiphanes quiteria quiteria* |  |  |  |  |  |  |  |  |  | 1 |  |  |
| *Pareuptychia summandosa* |  |  |  |  |  |  |  |  |  | 1 |  | 1 |
| *Posttaygetis penela* |  |  |  |  |  |  |  |  |  | 3 |  |  |
| *Pseudodebis marpessa* |  |  |  |  |  |  |  |  |  | 6 |  | 10 |
| *Pseudodebis valentina* |  | 1 |  | 3 |  |  |  |  | 1 | 4 | 1 | 11 |
| *Taygetis cleopatra* |  |  |  |  |  | 1 |  | 2 |  |  |  | 1 |
| *Taygetis mermeria* |  |  |  |  |  |  |  |  |  | 3 |  | 38 |
| *Taygetis rufomarginata* |  |  |  |  |  |  |  |  |  | 1 |  | 7 |
| *Taygetis thamyra* |  |  |  |  |  |  |  | 1 |  |  |  |  |
| *Taygetis virgilia* |  |  |  |  |  |  |  |  |  |  | 1 | 4 |
| **Total** | **4** | **13** | **10** | **38** | **9** | **19** | **5** | **16** | **9** | **32** | **19** | **111** |

Table S2. Multivariate homogeneity of groups dispersions (Betadisper) of fruit-feeding butterfly composition between stratum and season in each forest type. The analysis used the spatial median as a group mean, and p-values were based on 999 permutations.

|  | Terra firme | | Várzea | | Igapó | |
| --- | --- | --- | --- | --- | --- | --- |
|  | **F** | ***p*** | **F** | ***p*** | **F** | ***p*** |
| Stratum | 1.631 | 0.242 | 0.633 | 0.449 | 0.091 | 0.755 |
| Season | 0.2875 | 0.577 | 0.439 | 0.537 | 0.097 | 0.748 |


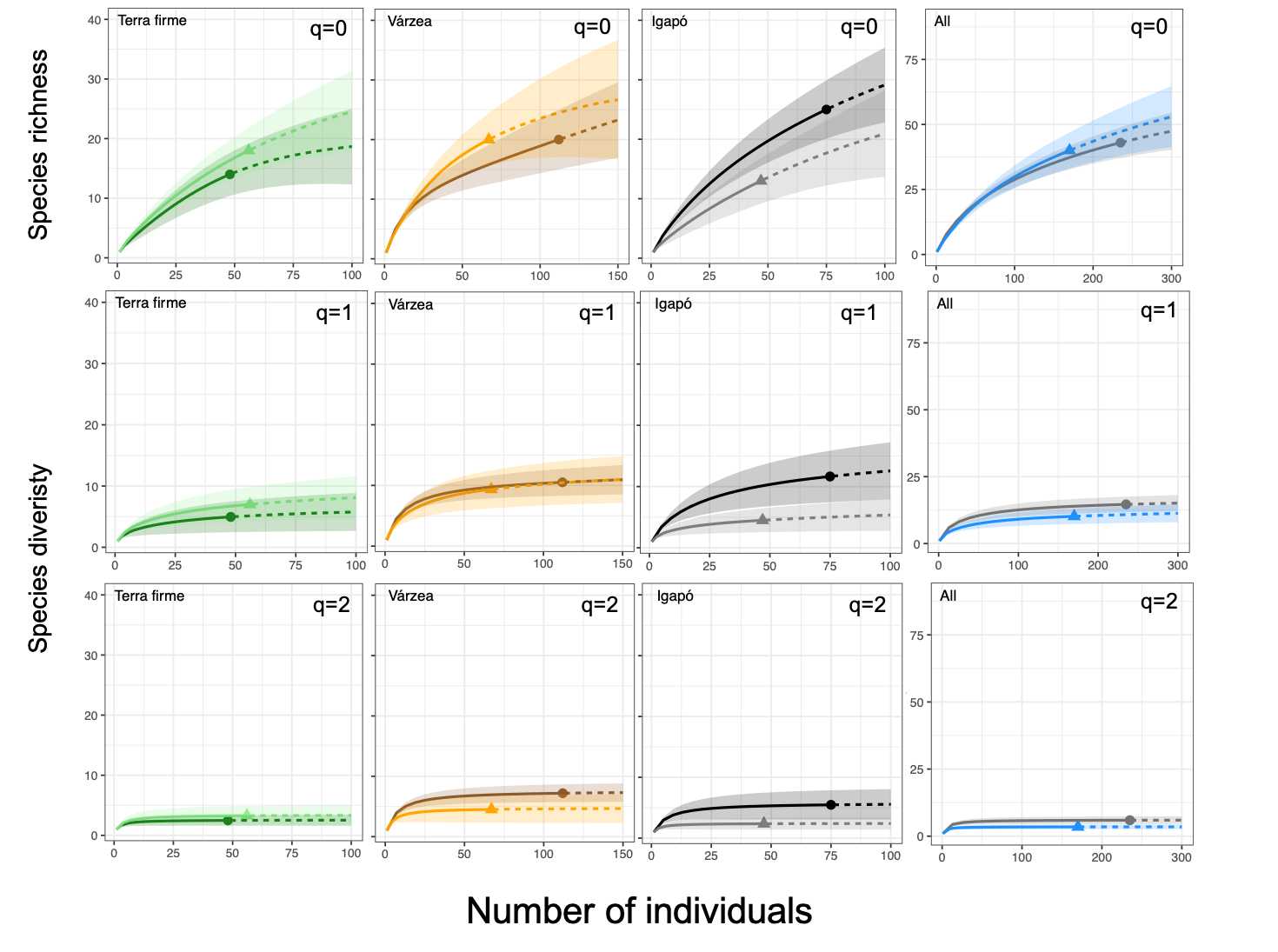


Fig. S1. Interpolation (solid line) and extrapolation (dashed line) curves in low- and high-water seasons (circles and triangles, respectively) using species richness, Shannon and Simpson diversities (qD = 0, 1, 2, respectively) of fruit-feeding butterfly assemblages of the combined data ("All") and butterfly assemblages of each forest type separately. Shaded areas represent 95% confidence intervals.


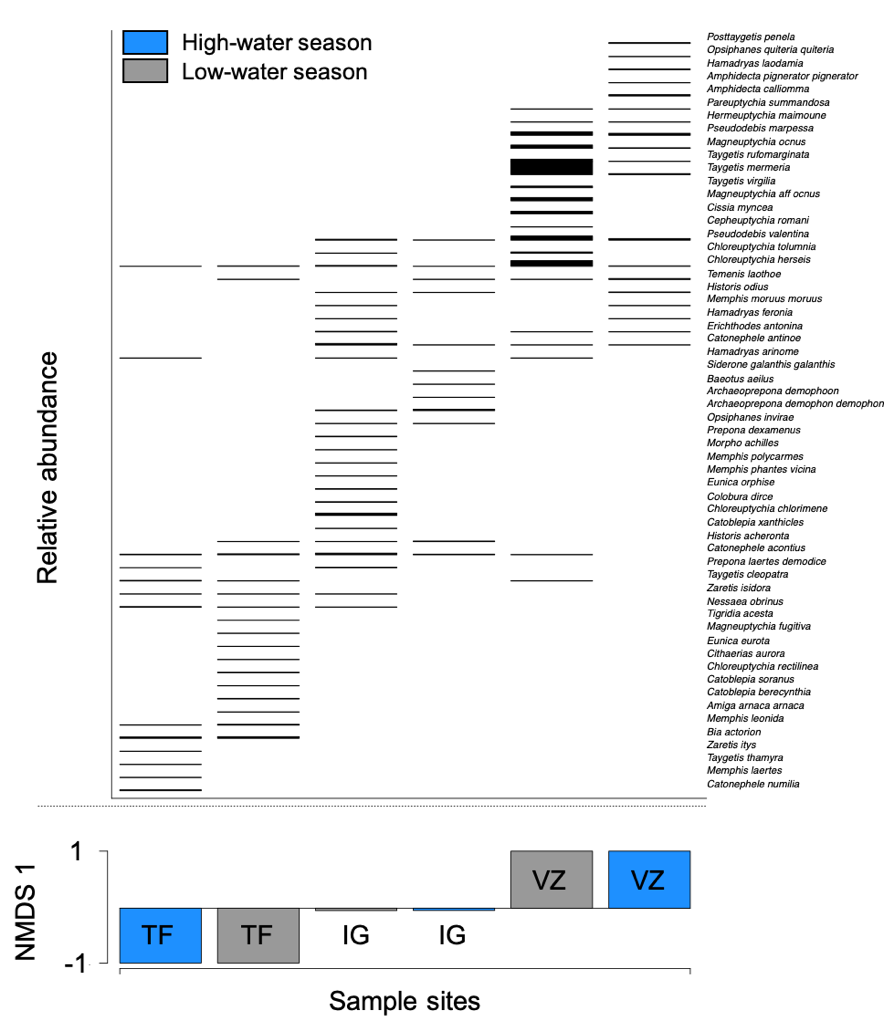


Fig. S2. Species distribution along the butterfly composition similarity gradient in each forest type and season. TF = *terra firme*, IG= *igapó*, VZ= *várzea*. ‘poncho’ function (Dambros, 2020).

Reference:

Dambros, C. S. (2020). csdambros/R‐functions: First release. https://github.com/csdambros/R–functions/tree/v1.0
